# Nanoemulgel Development of Stem Cells from Human Exfoliated Deciduous Teeth–Derived Conditioned Medium as a Novel Nanocarrier Growth Factors

**DOI:** 10.1055/s-0045-1806963

**Published:** 2025-04-23

**Authors:** Hendrik Setia Budi, Juni Handajani, Lisa Rinanda Amir, Sri Angky Soekanto, Ninik Mas Ulfa, Silvi Ayu Wulansari, Yung-Kang Shen, Shuntaro Yamada

**Affiliations:** 1 Department of Oral Biology, Dental Pharmacology, Faculty of Dental Medicine, Universitas Airlangga, Surabaya, Indonesia; 2 Cell and Biology Research, Surabaya Science Laboratory, Surabaya, Indonesia; 3 Department of Oral Biology, Faculty of Dentistry, Universitas Gadjah Mada, Yogyakarta, Indonesia; 4 Department of Oral Biology, Faculty of Dentistry, Universitas Indonesia, Jakarta, Indonesia; 5 Department of Pharmaceutica, Pharmacology and Clinical Pharmacy, Surabaya Pharmacy Academy, Surabaya, Indonesia; 6 School of Dental Technology, College of Oral Medicine, Taipei Medical University, Taipei, Taiwan; 7 Center of Translational Oral Research, University of Bergen, Bergen, Norway

**Keywords:** stem cells conditioned medium, growth factors, nanoemulgel, oral wound biotherapy, human well-being

## Abstract

**Objective** We aimed to develop a nanoemulgel of stem cells from human exfoliated deciduous teeth–derived conditioned medium (SHED-CM) for oral wound biotherapy candidate.

**Materials and Methods** Deciduous tooth pulp was collected from two patients aged 6 years. The mesenchymal stem cell marker expression was analyzed by immunocytochemistry of CD45, CD90, and CD105. Alizarin red staining was performed to differentiate SHEDs from osteoblasts. The quantitative and quantification of transforming growth factor-β (TGF-β) and vascular endothelial growth factor (VEGF) secreted into conditioned media were measured using sodium dodecyl sulfate polyacrylamide gel electrophoresis and enzyme-linked immunosorbent assay. The characteristics of the nanoemulgel of SHED-CM (NESCM) were analyzed in terms of organoleptic properties, pH, and homogeneity. The cytotoxicity of NESCM 1.5% was analyzed in human gingival fibroblast (hGF) cell and osteoblast cell line (MC3T3) by 3-[4,5-dimethylthiazol-2-yl]-2,5 diphenyl tetrazolium bromide assay.

**Statistical Analysis** The results were presented as mean ± standard deviation (X ± SD), and the differences between groups were analyzed using the post hoc Tukey's test at a significance level of *p-* value < 0.05.

**Results** SHEDs were successfully isolated, which were characterized for positive marker expressions of CD90 and CD105 and negative expression of CD45 as well as their osteogenic commitment. In SHED-CM, TGF-β and VEGF were detected on day 1 of conditioning and afterward. Notably, the growth factor enriched as the duration of conditioning increased. The generated nanoemulgel with SHED-CM was stable and homogeneous, and had limited cytotoxic effects on hGF and MC3T3 cell culture.

**Conclusion** SHED-CM containing the growth factors can potentially be used as oral wound biotherapy in the form of nanoemulgel.

## Introduction

The conditioned medium (CM) and secretome derived from mesenchymal stem cells (MSCs) exhibit considerable potential for advancing biotherapeutic approaches in the treatment of oral tissue diseases, particularly in biotherapy. 1 2 These cell-free treatment methods use bioactive molecules, including chemokines, cytokines, and growth factors, released by MSCs for promoting tissue regeneration and repair, 3 4 without the challenges associated with cell transplantation. 5 The growing field of research is creating opportunities to address a range of degenerative diseases through novel, minimally invasive methods. 6

In dentistry, CM from MSCs has been tested for regeneration of various tissues including pulp, dentin, bone, and periodontal tissues, 1 7 8 9 which was proven effective through its anti-inflammatory, 10 antiapoptotic, 11 antioxidative, 12 proangiogenic, 13 pro-osteogenic, and proneurogenic properties. 8 In particular, ∼30,000 proteins were identified in CM by dental pulp stem cells, 12 which significantly improved bone regeneration and angiogenesis *in vivo* . 14 Likewise, SHED-CM contains abundant secretome, enhancing its ability to regenerate soft and hard tissues. 15 Among these proteins, several studies have emphasized the existence and functions of crucial growth factors in SHED-CM, namely, transforming growth factor-β (TGF-β) and vascular endothelial growth factor (VEGF), which synergistically play a central role in soft and hard tissues regeneration and healing. 8 16 17 TGF-β controls cell specialization and extracellular matrix element production via the activation of intracellular signaling pathways, primarily the SMAD-dependent pathway, which regulates the transcription of target genes involved in cell proliferation, differentiation, and matrix deposition. 18 Additionally, TGF-β interacts with non-SMAD pathways, such as the MAPK and PI3K/Akt pathways, further influencing cellular behavior and promoting the synthesis of key extracellular matrix proteins such as collagen and fibronectin, which are essential for tissue regeneration and repair. 19 On the contrary, VEGF has a crucial function in stimulating angiogenesis by facilitating endothelial cell growth, migration, and lumen formation. 20 MSC-CM is known to include these growth factors, and their application may potentially resolve complex dental diseases that regular treatments may not adequately manage. 21 However, although the MSC-derived CM shows promise, the application in regenerative medicine and dentistry requires careful consideration in CM production and cell-type-specific optimization. 4 These include the selection of cell types, passages, 22 and production protocol in a serum-free condition. While much is in common among MSC-derived CM, dental stem cells-derived CM stands out for rich neurotrophic factors with a higher antioxidant capacity due to their neural crest origin compared with bone marrow stem cells- or adipose tissue stem cells-derived CM. 23 Growth factor levels may also fluctuate depending on a conditioning period due to the dynamic enrichment and biodegradation of the secreted molecules. The complex nature of the CM makes it challenging to standardize CM production and predict outcomes. 24 25

CM by MSC contains lipophilic and hydrophilic substances, which can be dissolved, diluted, and degraded due to the presence of saliva in oral cavity. 26 Furthermore, retention of CM for prolonged effects of the bioactive molecules is crucial for successful tissue repair and regeneration. Thus, CM is often combined with various carriers including hydrogels to promote sustainable bioactivity. In tissue engineering, nanoemulsion-based gel (i.e., nanoemulgel) has emerged as a carrier, offering a versatile platform for delivering bioactive molecules, drugs, and growth factors to promote tissue regeneration. 27 These hybrid systems combine the unique properties of nanoemulsions—such as high solubility, stability, and enhanced bioavailability of hydrophobic compounds—with the structural advantages of gels, including ease of application and prolonged retention at the target site. Nanoemulgels can serve as carriers for therapeutic agents, facilitating controlled and localized release to modulate cellular behavior and accelerate healing processes. Their nanosized droplet structure enables efficient penetration into tissues, while the gel matrix provides a scaffold-like environment, potentially supporting cell attachment and proliferation. 28 Moreover, nanoemulgels can be customized with biocompatible and biodegradable components, making them suitable for a wide range of regenerative applications, including bone, cartilage, and soft tissue repair. However, the use of nanoemulgel as a carrier of the CM and its biocompatibility has not yet explored. Therefore, this study aimed to develop a nanoemulgel of SHED-CM (NESCM) aiming for oral wound biotherapy. The study evaluated the preparation method and bioactive components of SHED-CM followed by biocompatibility testing of NESCM.

## Materials and Methods

### Ethical Clearance

This study was conducted in accordance with the ethical principles, which included acquiring written informed consent from the donors and obtaining ethics approval from the Committee for Medical and Health Research Ethics at the Faculty of Dental Medicine (0530/HRECC.FODM/V/2024). Deciduous teeth were extracted from two 6-year-old patients in the surgery room using the aseptic technique. The criteria for the tooth sample are anterior tooth without carries, pulp that appears red in color, no infection on the gum, and the tooth is extracted in an aseptic condition. 29

Teeth were rinsed with 5% penicillin/streptomycin (PenStrep; P4333, Sigma-Aldrich, St. Louis, Missouri, United States) in phosphate-buffered saline (PBS; 806544, Sigma-Aldrich) and immediately transported to the cell laboratory on an ice box at 4°C.

### SHED Isolation and Cell Expansion

Pulp tissues from deciduous teeth were enzymatically digested using collagenase type I (4 mg/mL) and dispase (2 mg/mL) for 1 hour at 37°C before being plated into a dish cell culture. SHEDs were cultured in a 35-mm dish (CLS430165, Corning Inc., Corning, New York, United States) with a growth medium comprising α-minimum essential medium Eagle (α-MEM; M4526, Sigma-Aldrich) supplemented with 10% fetal bovine serum (FBS; F9665, Sigma-Aldrich) and 1% penicillin/streptomycin at 37°C and 5% CO _2_ in a humidified environment. The growth medium was changed every 2 to 3 days and subcultured. SHEDs at passage 2 were used for the surface marker characterization and differentiation analysis, and passage 4 for producing SHED-CM. 30 To determine the presence of contaminants and cell development when the medium was changed, the cells were routinely observed under an inverted microscope.

### MSC Surface Marker Characterization

Immunocytochemistry was employed to analyze the expression of CD45, CD90, and CD105, which are standard markers for MSCs. 31 SHEDs at passage 2 were plated at 12,500 cells/well into a 35-mm disk with a complete medium. When cells reached 70 to 80% confluence, the medium was removed and washed with PBS. Following overnight attachment, the cells were fixed in 4% paraformaldehyde (PFA; 1.00496, Sigma-Aldrich) and stored in 70% ethanol at 4°C until staining. Primary and fluorophore-conjugated secondary antibodies were diluted in a blocking solution and incubated at 4°C overnight following the product protocol of antihuman CD45 antibody (100–0316; Stemcell Technology, United States), CD90 (60045; Stemcell Technology), and CD105 (60039BT; Stemcell Technology).

### Osteogenic Differentiation Analysis by Alizarin Red Staining

SHEDs at passage 2 were plated at 12,500 cells/well into a 35-mm disk and cultured in a complete medium at 37°C and 5% CO _2_ in a humidified environment. When cells reached 70 to 80% confluency, the medium in the induction group was changed to the OsteoMax-XF osteogenesis differentiation kit (SCM121; Sigma-Aldrich), whereas the control group was continuously grown in the growth medium. At days 14 and 21, the samples were washed in PBS and subsequently fixed in 4% PFA for 40 minutes. The cell was thoroughly washed with distilled water. The mineral deposition was stained with 0.1% Alizarin Red S (A5533; Sigma-Aldrich) for 20 minutes and subsequently washed six times with distilled water. The mineralized nodule formation was observed under a microscope. 31

### SHED-CM Isolation

To produce SHED-CM, cells from donors at passage 4 were inoculated at a density of 2 million cells into a T-75 flask and grown in the complete growth medium. Upon achieving 70 to 80% confluency, the cells were rinsed with PBS twice and subsequently changed the medium with serum-free α-MEM. The cells were further incubated at 37°C and 5% CO _2_ in a humidified environment for 1, 3, and 5 days to collect SHED-CM. This equates to ∼15 mL of CM by 8 to 10 million SHEDs. Following CM collection, centrifugation was performed at 1,200 rpm for 5 minutes to isolate the CM, which was subsequently filtered through a 0.2-μm-pore-size filter and kept at −80°C. The SHED-CM collected on day 1 (SCM-H1), day 3 (SCM-H3), and day 5 (SCM-H5) were stored for the next analysis.

### Protein Quantification of SHED-CM Using Sodium Dodecyl Sulfate Polyacrylamide Gel Electrophoresis

This study used SHED-CM which stored previously for this analysis. To quantify the proteins secreted into the CM was performed by sodium dodecyl sulfate polyacrylamide gel electrophoresis (SDS-PAGE) on a 10% separating gel. 32 First, we prepared the sample buffer and heated it at 70°C for 5 minutes using a water bath. Next, we placed the protein sample SHED-CM (i.e., SCM-H1, SCM-H3, and SCM-H5), whose protein concentrations were measured using a NanoDrop spectrophotometer (NanoDrop One; ThermoFisher, Germany), into wells containing the sample buffer. Electrophoresis was performed at a constant current of 150 mA for 80 minutes. Subsequently, the distribution of the bands was detected using Coomassie Brilliant Blue gel staining G-250 (LC6065; ThermoFisher, United States). The molecular weight of the protein was determined by inserting the migration distance into the standard marker (M00624S; GenScript, Piscataway, New Jersey, United States) linear regression equation.

### Enzyme-Linked Immunosorbent Assay-Based Quantification of TGF-β and VEGF Levels in SHED-CM

This study used SHED-CM, which was stored previously for this analysis. The five groups were performed, including the basal medium (BM) as a negative control, wherein cells were expanded in α-MEM; a positive control group, wherein cells were expanded in α-MEM and 10% FBS; SCM-H1; SCM-H3; and SCM-H5. Human TGF-β (BZ-08124310-EB; Bioenzy, Taiwan) and VEGF (BZ-08120800-EB; Bioenzy, Taiwan) enzyme-linked immunosorbent assay (ELISA) kits were used to quantify the growth factors in SHED-CM (SCM-H1, SCM-H3, and SCM-H5) in accordance with the manufacturer's protocols. All conditions were tested as triplicates.

The TGF-β levels were quantified using a standard curve by the following equation:

*Y* = 0.0005 *x* + 0.1123

*R*^2^ = 0.9886

Likewise, the VEGF levels were measured using a standard curve by the following equation:

*Y* = 0.0004 *x* + 0.1385

*R*^2^ = 0.9879

### Fabrication and Characterization of the NESCM

Carboxymethyl cellulose sodium (CMC-Na; C4888, Sigma-Aldrich) with hot water was prepared to produce 1.5 and 1.8% of CMC into separate mucilago in a mortar. After 15 minutes of development and stirring, polyethylene glycol (52887; Sigma-Aldrich) was gradually added until a homogenous gel mass was produced. The resulting gel was chilled for 45 to 60 minutes at 4°C. It was subsequently transformed into a nanogel using a thorax technique, which involved ultracentrifugation at 11,000 rpm for 10 minutes. To generate an emulgel, the prepared CM was added to the nanogel at 4°C. Next, it was mixed until homogenous, and the process of generating the nanoemulgel was continued by stirring at 1,000 rpm for 15 minutes to ensure that the secretome was uniformly distributed in the emulgel precursors. The entire process of making the nanoemulgel preparations was conducted in a sterile room and performed aseptically in laminar air flow. 33 Furthermore, the nanoemulgel formed was stored in a refrigerator to perform the particle size analysis (PSA), polydispersity index (PDI), and zeta potential evaluation. To determine the droplet size, PDI, and zeta potential in the nanoemulgel, the Zetasizer Nano ZS apparatus (Malvern PCS Instruments, Malvern, UK) was employed. The nanoemulgel was distributed inside the device's disposable-sized cuvette cell. The hydrodynamic diameter and PDI of the nanoemulgel droplets were measured using photon correlation spectroscopy at 20°C and a scattering angle of 173°C. The Malvern Zetasizer equipment measures the fluctuation in light scattering caused by the Brownian motion of the particles over time. 34

### Cytotoxicity Evaluation of the NESCM

The primary cells from the human gingival fibroblast (hGF) at passage 5 from the Archived Biological Material of Stem Cell Research Center, Faculty of Dental Medicine, Universitas Airlangga and the MC3T3 osteoblast cell line from the American Tissue Culture Collection (Manassas, Virginia, United States) were used for cytotoxicity screening. The hGF cells were cultured in a complete medium of Dulbecco's modified Eagle medium and Ham's F12 Nutrient Mixture (DMEM/F1; D9785, Sigma-Aldrich), whereas the MC3T3 cells were cultured in DMEM High Glucose (D5796, Sigma-Aldrich). In both cell culture media, 10% FBS (F9665; Sigma-Aldrich) and 1% antibiotic and antimycotic (penicillin 100 U/mL, streptomycin 100 μg/mL, and amphotericin B 0.25 μg/mL; Sigma-Aldrich) were added. The cells were seeded in 75-cm ^2^ flasks (Costar; Corning Inc., Corning, New York, United States) under culture conditions of 37°C and 5% CO _2_ , and the medium was changed at 2 to 3-day intervals until confluence. The cells were detached by trypsinization using a 0.05% trypsin-EDTA solution (Sigma-Aldrich) at 80 to 90% confluence and subsequently subcultured onto 96-well plates at 10,000 cells density per well with 100 μL of complete medium and incubated for 24 hours at 37°C and 5% CO _2_ for cell adhesion. 35

We tested eight groups, including a control medium (CM; BM without cells), control cell (CS; cells with complete medium), SCM-H1, SCM-H3, SCM-H5, NESCM-H1, NESCM-H3, and NESCM-H5. When cells reach 70 to 80% confluence in 96-well plate, the culture medium was replaced with test media in the total volume of 100 μL in which the ratio of the fresh medium and sample was 50:50 in the treatment groups (SCM-H1, SCM-H3, SCM-H5, NESCM-H1, NESCM-H3, and NESCM-H5). The cells were incubated for 24 hours at 37°C and 5% CO _2_ . All of the groups were tested in triplicate wells. The viability of each well plate was assessed by adding a 3-[4,5-dimethylthiazol-2-yl]-2,5-diphenyl tetrazolium bromide (MTT) solution of 50 μL (5 mg/mL) for 4 hours, and it was stopped by adding 50 μL of dimethyl sulfoxide (D4540, Sigma-Aldrich). After 30 minutes, the absorbance was measured using a spectrophotometer (Epoch BioTex, Winooski, Vermont, United States) at a 570-nm wavelength. 35 The absorbance levels were quantified using the following equation:



Where abs is the absorbance; abs CM is the absorbance of control medium; and abs CS is the absorbance of control cell.

### Statistical Analysis

The levels of TGF-β and VEGF were tabulated and analyzed using the Statistical Package for the Social Sciences (SPSS) with a one-way analysis of variance test at a 95% confidence level. The results were presented as mean ± standard deviation (X ± SD). Furthermore, differences between groups were analyzed using the post hoc Tukey's honest significant difference test at a significance level of *p-* value < 0.05.

## Results

### Isolation and Cell Expansion of SHED

**Fig. 1:**
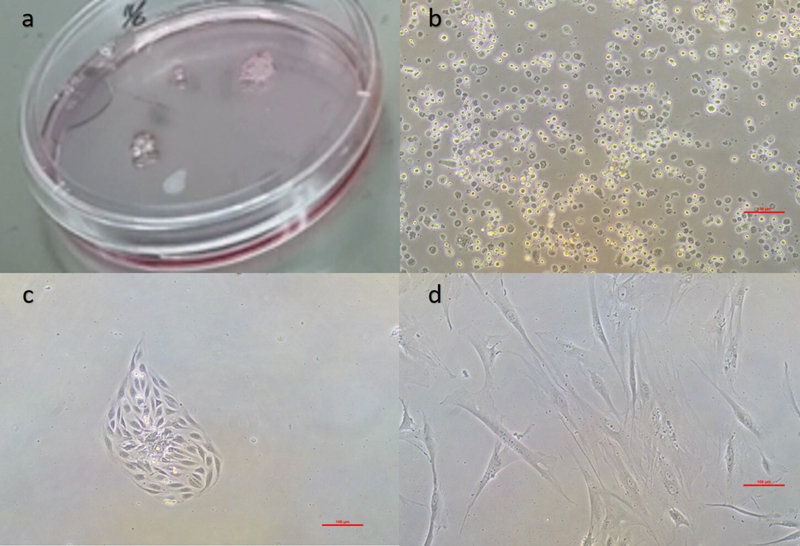
Monitoring SHED morphology under an inverted microscope to confirm cell health and growth characteristics during culture. (a) Pulp tissues from deciduous teeth are enzymatically digested and seeded in fresh medium; (b) image of SHED isolation on day 0 under an inverted microscope; (c) initial growth of SHED on day 7; and (d) low-density growth of SHED exhibiting fibroblast-like morphology on day 20. SHED, stem cells from human exfoliated deciduous teeth.

This study successfully obtained SHED from a 6-year-old patient donor. SHEDs showed a fibroblast-like spindle-shaped morphology ( Fig. 1A–D ). The SHEDs were elongated and showed tapering at both ends, which is a frequent feature. The *in vitro* growth and expansion of SHED cells typically preserves their homogeneous shape, with a more pronounced spindle-like appearance by the fourth passage. This morphology is crucial for determining the mesenchymal lineage of the cells and is frequently linked to their capacity to differentiate into diverse cell types, including osteoblasts.

### MSCs from SHED Exhibited Expression on Surface Marker to CD90 and CD105

**Fig. 2:**
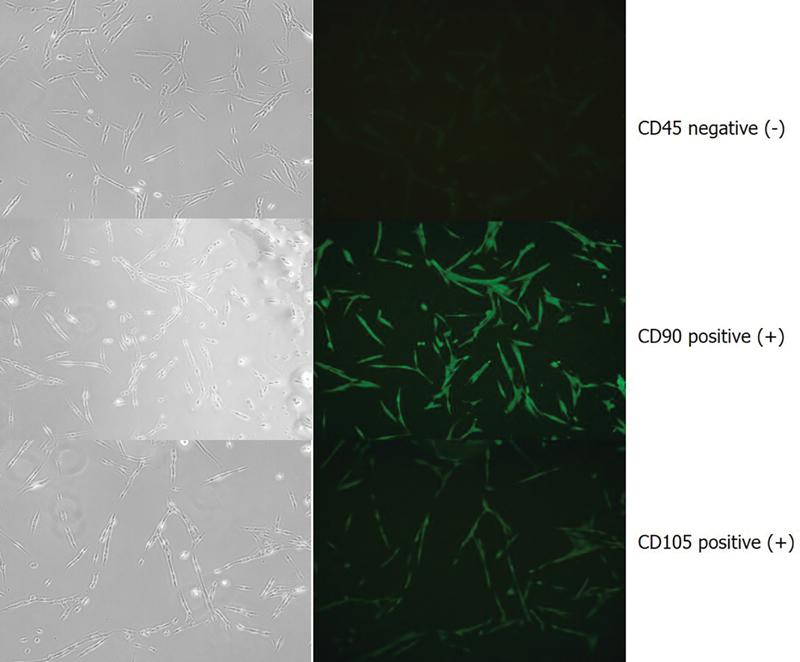
SHED isolation demonstrating that mesenchymal cells are positive for CD90 and CD105 through immunocytochemical analysis. SHED, stem cells from human exfoliated deciduous teeth.

SHED passage 2 from the patients ubiquitously expressed CD90 and CD105 as a positive MSC surface marker, whereas CD45 expression was not observed. The negative CD45 expression distinguishes SHEDs from hematopoietic cells ( Fig. 2A–C ).

### Osteogenic Differentiation Analysis Using Alizarin Red Staining

**Fig. 3:**
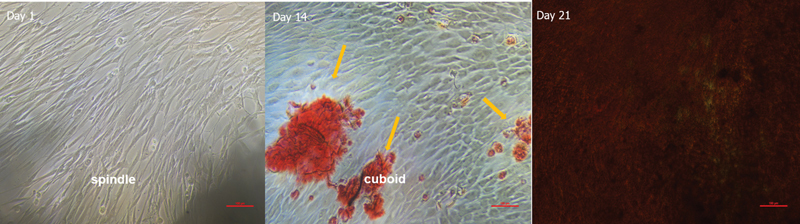
Alizarin red as a mineralization marker for SHED culture. (Day 1) Fibroblast-like cell morphology (spindle-shaped); (day 14) fibroblast-like cell morphology (spindle-shaped) differentiates into osteoblast-like cells (cuboidal). The yellow arrow indicates mineralization nodules; (day 21) cells completely covered by the mineralized nodule formation. SHED, stem cells from human exfoliated deciduous teeth.

The SHED at passage 4 exhibited the differentiation from fibroblast-like cells to osteoblast-like cells under the osteoinductive condition. The presence of calcium deposits was visualized by the Alizarin Red S staining ( Fig. 3 ). On day 14, the mineralized nodule formation began to appear, depositing calcium and phosphate into the extracellular matrix. On day 21, the accumulation of the mineral deposit was robustly visualized.

### Isolated and Selected Growth Factor Analysis of SHED-CM Using SDS-PAGE

SHED-CM was prepared with three different incubation periods applied (i.e., SCM-H1, SCM-H3, and SCM-H5). The total protein in the SHED-CM measured using a NanoDrop spectrophotometer is shown in Table 1 . Interestingly, SCM-H1 and SCM-H5 groups contained significantly higher protein quantities compared with SCM-H3 ( *p* < 0.05), suggesting the incubation period is a determinant for protein concentration in CM.

**Table 1:** Analysis of protein concentration in stem cells from human exfoliated deciduous teeth-conditioned medium on days 1, 3, and 5 using a NanoDrop spectrophotometer

Sample	Protein concentration (ug/mL)	*p* -Value
SCM-H1	161 ± 12.1 ^a^	
SCM-H3	44 ± 3.7 ^b^	0.000
SCM-H5	153 ± 6.4 ^b,c^	

Abbreviation: SCM, stem cells from human exfoliated deciduous teeth–derived conditioned medium.

Note: Different superscript letters indicate a significant difference ( *p* < 0.05).

**Fig. 4:**
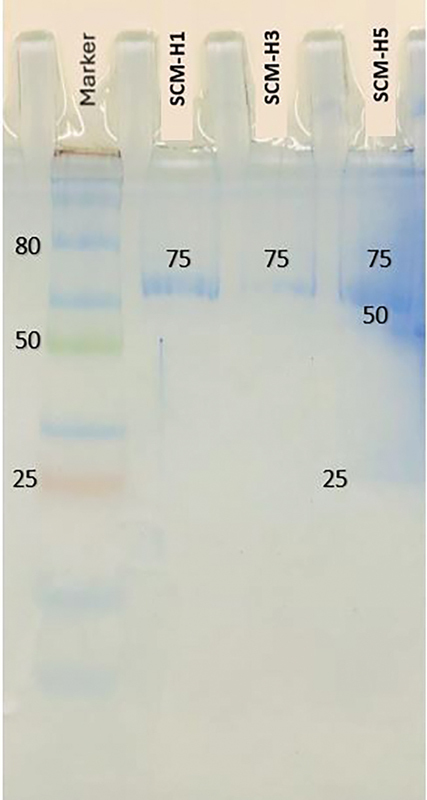
Analysis of the growth factors using sodium dodecyl sulfate polyacrylamide gel electrophoresis (SDS-PAGE) on stem cells from human exfoliated deciduous teeth–derived conditioned medium collected on days 1 (SCM-H1), 3 (SCM-H3), and 5 (SCM-H5). The molecular weight of transforming growth factor-β was detected at 25 kDa, VEGF at 50 kDa, and hepatocyte growth factor at 75 kDa in SCM.

SDS-PAGE analysis of the molecular weight band of the growth factors present in SCM-H1, SCM-H3, and SCM-H5 was performed. The present study predicted that the band at a molecular weight of 75 kDa corresponds to the hepatocyte growth factor (HGF). Although it appeared faint on day 3, it became more prominent on days 1 and 5. This observation aligns with the protein concentration measurements obtained using NanoDrop, which indicated reduced total protein on day 3. On day 5, three bands were detected with molecular weights of 27, 50, and 75 kDa. This study hypothesized that the 27-, 50-, and 75-kDa bands represent TGF-β, VEGF, and HGF, respectively ( Fig. 4 ).

### ELISA-Based Quantification of Selected Growth Factors in SHED-CM

**Fig. 5:**
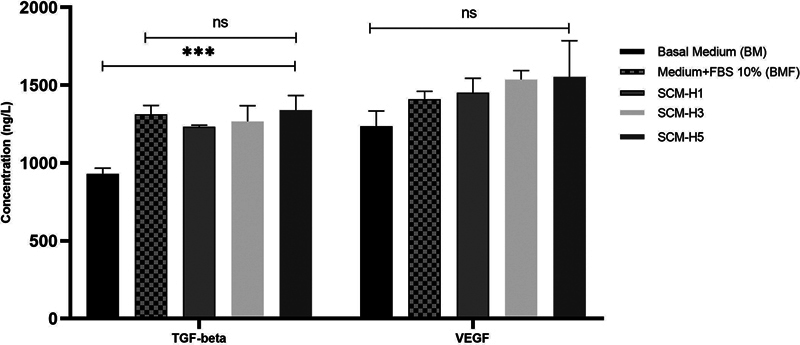
Concentration analysis of transforming growth factor-β (TGF-β) and vascular endothelial growth factor (VEGF) in SHED-CM isolation at different time points using enzyme-linked immunosorbent assay. The levels of TGF-β significantly increased ( *p* = 0.000) on each day of isolation compared with the basal medium; however, no significant increase in VEGF levels was noted ( *p* = 0.107). ***, statistically highly significant at *p* = 0.0001; ns, no significant differences.

The levels of TGF-β and VEGF were quantified in the BM, complete medium with supplement (BMF), SCM-H1, SCM-H3, and SCM-H5 groups. After 1 day of conditioning, the TGF-β level reached ∼1,200 ng/L, gradually increasing until 5 days in a time-dependent manner ( Fig. 5 ). The TGF-β levels in SHED-CM comparable to the serum-supplemented control medium were significantly higher than those without serum (BM) at *p* -value < 0.05. However, these three groups did not show a significant difference from the BMF group ( *p* > 0.05). Likewise, the VEGF levels in the SHED-CM group consistently increased over the conditioning periods from 1 to 5 days despite no statistical significance ( *p* > 0.05), mostly due to small sample numbers ( Fig. 5 ). The highest VEGF level was observed at ∼1,500 ng/L on day 5 (SCM-H5).

### Fabrication and Characterization of the NESCM

The preparation of nanoemulgel formulations with 1.5 and 1.8% CMC-Na concentrations was intended for application on both soft and hard tissue wounds. The NESCM formulation was evaluated for organoleptic properties, pH, homogeneity, and particle size (PSA). SCM-H1, SCM-H3, and SCM-H5, incorporated in the nanogel bases of 1.5 and 1.8%, exhibited an initial increase in the pH level; however, no significant difference was observed ( *p* = 0.892) among the groups. Homogeneity testing, based on visual and subjective parameters, confirms the absence of coarse particles in the NESCM-H1, NESCM-H3, and NESCM-H5 formulations, indicating that the samples are homogeneous, as outlined in the NESCM formulation characterization results in Table 2 .

**Table 2:** Characterization of nanoemulgel of SHED-CM through organoleptic visualization, pH level measurement, and homogeneity assessment

Groups	Organoleptic	pH	Homogeneity
1.5% nanoemulgel bases	Clear, odorless, gel, no sediment	7.68	Homogenous
SCM-H1 + 1.5% nanoemulgel bases	Clear, odorless, gel, no sediment	8.51	Homogenous
SCM-H3 + 1.5% nanoemulgel bases	Clear, odorless, gel, no sediment	8.44	Homogenous
SCM-H5 + 1.5% nanoemulgel bases	Clear, odorless, gel, no sediment	8.69	Homogenous
1.8% nanoemulgel bases	Clear, odorless, gel, no sediment	8.16	Homogenous
SCM-H1 + 1.8% nanoemulgel bases	Clear, odorless, gel, no sediment	8.36	Homogenous
SCM-H3 + 1.8% nanoemulgel bases	Clear, odorless, gel, no sediment	8.53	Homogenous
SCM-H5 + 1.8% nanoemulgel bases	Clear, odorless, gel, no sediment	8.53	Homogenous

Abbreviation: SCM, stem cells from human exfoliated deciduous teeth–derived conditioned medium.

**Fig. 6:**
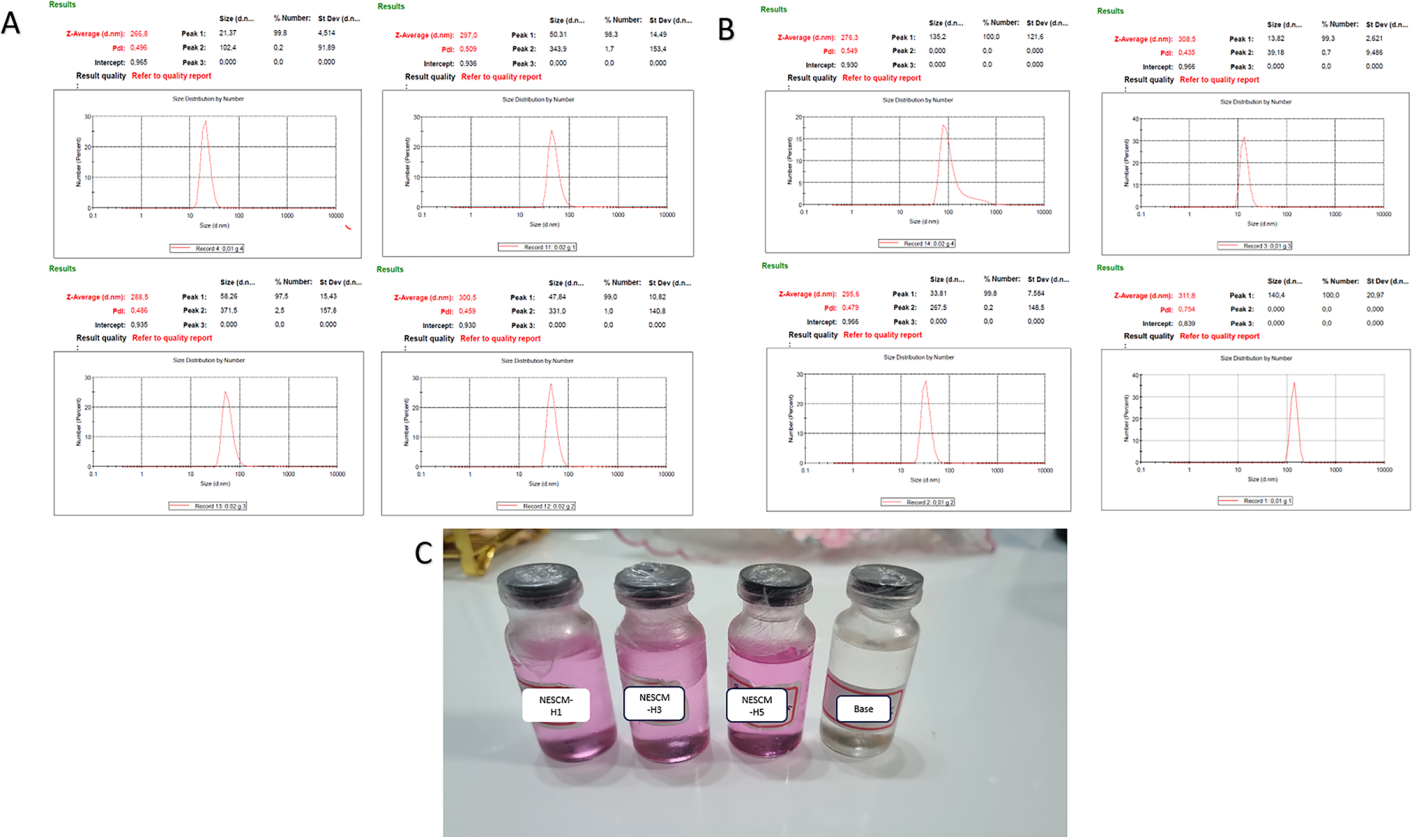
Characteristic of nanoemulgel SCM formulations (NESCM). Particle size distribution of NESCM in 1.5% formulation (A) and 1.8% formulation (B). The data of particle size distribution were presented in nanometer (nm). Preparation of nanoemulgel formulations of SCM in carboxymethyl cellulose sodium (C).

The particle size measurements of NESCM at 1.5 and 1.8% indicate that the particle size distribution is within the nanoparticle range, as shown in Fig. 6 . The 1.5% (266.8 nm) and 1.8% (276.3 nm) basal groups showed an increase of ∼8 to 13% and 7 to 13% in the particle size distribution, respectively. The NESCM composition included not only CMC-Na particles but also other particles secreted within the SCM.

### Cytotoxicity Evaluation of the NESCM

**Fig. 7:**
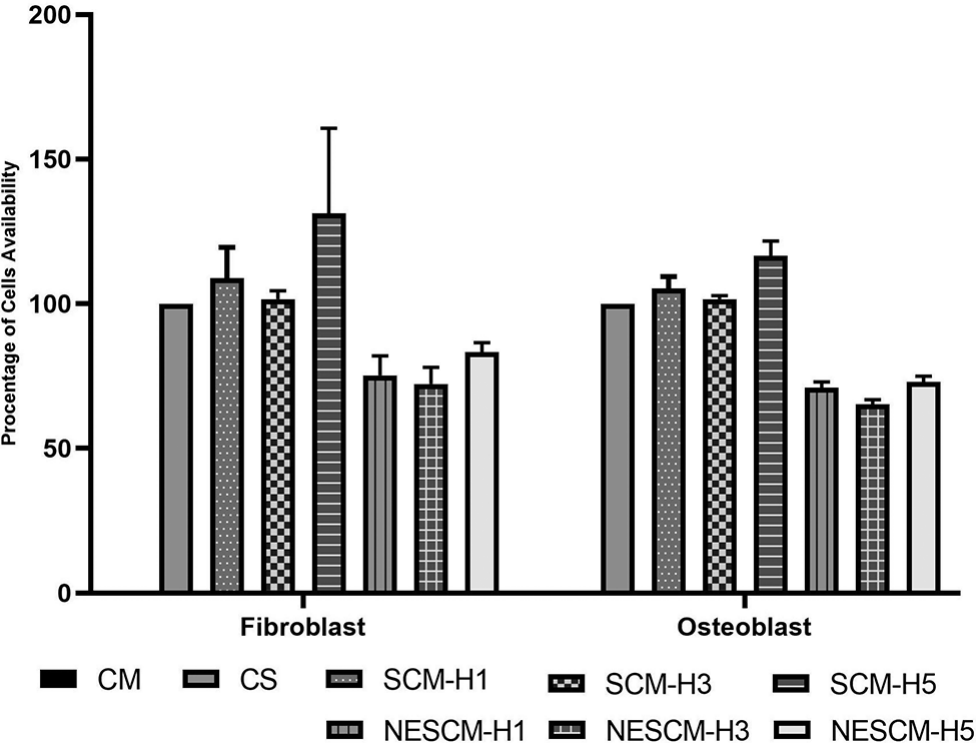
Nanoemulgel 1.5% formulation of SHED-CM and cytotoxicity assessment on hGF and MC3T3 cell culture. The lowest cell viability being 72% in fibroblasts and 65% in osteoblasts. CM, control medium without cell; CS, control cell with cells; hGF, human gingival fibroblast; SCM-H1, SHED-CM collected on day 1; SCM-H3, SHED-CM collected on day 3; SCM-H5, SHED-CM collected on day 5; NESCM-H1, nanoemulgel of SHED-CM collected on day 1; NESCM-H3, nanoemulgel of SHED-CM collected on day 3; NESCM-H5, nanoemulgel of SHED-CM collected on day 5.

SHED-CM sustained or improved the viability of hGF and MC3T3 compared with the control cell group (BMF), as shown in Fig. 7 . Interestingly, SCM-H5 supported the highest cell growth and viability compared with SCM-H1 and SCM-H3. However, the application of NESCM to primary hGFs significantly reduced the fibroblast viability compared with both the SCM and control cell groups ( *p* < 0.001). On average, the fibroblast viability in the NESCM group remained > 72%.

Compared with the control cell group (BMF), osteoblast viability in the SCM-H1–, SCM-H3–, and SCM-H5–treated groups increased. The NESCM-H5 group had higher osteoblast viability than the NESCM-H1 and NESCM-H3 groups. The use of NESCM in MC3T3 significantly reduced viability compared with the groups treated with SHED-CM (SCM-H1, SCM-H3, and SCM-H5) and the BMF ( *p* < 0.001). On average, the osteoblast viability in the NESCM group was > 65%.

The cytotoxicity of NESCM 1.8% could be presented in this study. The gelling weight might affect cell detachment when the gel is applied to each well in the NESCM group. The cell floated on the surface of cell medium at 1.8% nanoemulgel concentration.

## Discussion

SHEDs possess extraordinary therapeutic potential in regenerative medicine for their multipotency and abundant secretome profile. 36 The results of our study revealed that the total protein in SCM gradually fluctuated upon isolation, and higher levels were observed in SCM-H5 using NanoDrop. Several biological and experimental factors can influence the substance fluctuations within SCM. SHED is a type of MSC known for secreting various bioactive molecules. The CM composition is one of the key aspects influenced by primary cell passage, and the collection of growth factors, cytokines, and other bioactive molecules were secreted by the stem cells into the medium. 8 SCM isolation at passage 4 detected growth factors, including TGF-β at 27 kDa of molecular weight and VEGF at 50 kDa, in a prolonged incubation of 5 days following cell confluence but were not detected on days 1 and 3 of incubation. However, HGF at 75 kDa was consistently detected from day 1 following cell confluence until day 5 using SDS-PAGE. The molecular weight of HGF at 75 kDa is similar to HGF human recombinant. 37 These growth factors are crucial for promoting tissue repair and regeneration by stimulating cell proliferation, migration, and angiogenesis. Growth factors, including TGF-β and VEGF, are a significant component of the secretome produced by MSCs and play a crucial role in their therapeutic potential. The presence of HGF in SCM-H1, SCM-H3, and SCM-H5 enhances the overall regeneration capacity by promoting wound healing, decreasing inflammation, and enabling tissue repair. The capacity of SHED to release HGF enhances its therapeutic potential in various regenerative medicine applications, notably oral tissue regeneration.

The SDS-PAGE analysis of TGF-β and VEGF in SCM requires validation by measuring their levels using the ELISA method. Compared with SCM on days 1 and 3, the TGF-β and VEGF levels in SHED-CM isolated on day 5 (SCM-H5) were the highest. This finding supports the SDS-PAGE findings, wherein TGF-β and VEGF bands were only detected on day 5. Although TGF-β and VEGF bands were not detected in SCM-H1 and SCM-H3, the levels of these growth factors were detectable using ELISA, indicating that ELISA is effective for detecting growth factors at minimal levels. ELISA and SDS-PAGE are frequently used techniques for detecting and quantifying proteins, including growth factors. However, they significantly differ in their sensitivity and specificity. Generally, ELISA is considered to be more sensitive than SDS-PAGE in terms of growth factor detection. This is because of the enzymatic amplification step involved in ELISA, which can significantly magnify the signal, thereby enabling the detection of very low concentrations of the target protein. 38 When using SDS-PAGE for detecting proteins in CMs, a specific protein concentration in the sample is necessary for the visible band formation. The measured TGF-β and VEGF levels in SCM were higher than those in the BM, indicating that the stem cells in the SHED culture are actively secreting these growth factors into the CM. This finding indicates that SHEDs are a source of TGF-β and VEGF. SHEDs could be a valuable TGF-β and VEGF source for therapeutic applications, especially in conditions wherein these growth factors are deficient. These findings provide insights into the biology of SHEDs and their role in growth factor production and regulation. Furthermore, the TGF-β and VEGF levels within the SCM were comparable to those in the complete medium (BMF), and SHEDs produced similar TGF-β and VEGF levels as FBS. It is likely that the stem cells in the SHED culture are naturally producing TGF-β and VEGF at concentrations comparable to those detected in FBS, which could indicate that SHEDs are a rich source of these growth factors.

Our findings from the abovementioned data support the argument that lower passage numbers are generally optimal for producing a rich CM in the growth factors necessary for effective biotherapy. Growth factor concentration will increase after several days of incubation. Early passages, such as passages 3 to 5, are preferred as they maintain higher levels of essential growth factors, which are crucial for regenerative processes. As the passage numbers increased, a decline in the secretion of these vital components is noted, reducing the therapeutic potential of the CM. The fourth passage of MSCs can be considered a stable point for isolating the CM; however, evaluating the specific context and requirements of the intended application is crucial.

For SHED-CM to effectively penetrate mucosal tissues, selecting an appropriate drug delivery system is imperative. The use of an emulgel as a vehicle for SCM is justified by its demonstrated superior absorption capabilities compared with conventional gels in the context of CM application. 39 SCM can encompass both lipophilic and hydrophilic compounds, contingent upon the specific biomolecules secreted by the cells. Lipophilic substances, characterized by their affinity for lipids and solubility in nonpolar solvents, include molecules such as steroid hormones, fatty acids (e.g., arachidonic acid), lipid-soluble vitamins (e.g., vitamin D), and certain growth factors such as TGF-β. Additionally, the lipophilicity of the polymer may reduce the capacity of VEGF to become entrapped, potentially leading to a rapid release. Conversely, hydrophilic substances, which are soluble in water, comprise proteins, sugars, and amino acids, which are integral to various cellular functions.

The penetration of growth factors in SHED-CM using an emulgel formulation for oral cavity applications can be enhanced by transforming it into a nanoemulgel. 27 The nanoemulgel formulation of the NESCM possesses a nanoscale structure that facilitates deeper penetration into the mucosal layers, thereby increasing its therapeutic efficacy. Furthermore, the nanoemulgel structure contributes to the improved physical stability. This formulation protects the active ingredients from enzymatic degradation and hydrolysis. The emulsification achieved through this emulgel formulation is highly effective for dispersing active substances and remains stable, ensuring that significant efficacy in oral mucosal therapy is maintained by SCM. Surfactants, including propylene glycol, in the nanoemulgel can enhance mucosal tolerance and reduce irritation, thereby improving user comfort, as the formulation must withstand dissolution or flow easily. Therefore, the SHED-CM–derived nanoemulgel offers advantages in terms of penetration, physical stability, drug protection, controlled release, and favorable biocompatibility and mucosal tolerance.

To detect and characterize particles in the SHED-CM, particle size analyzers can be employed. The particle composition in SCM can widely vary depending on the cell type used for conditioning it, the culture conditions, and the collection and storage methods. Exosome vesicles (30–150 nm), microvesicles (100–1,000 nm), cell debris, protein aggregates, and lipid droplets are some common particle types. However, of note, particle size analyzers may not be able to distinguish between different particle types on the basis of their composition. These findings confirm that the particle size of NESCM is greater than that of the gelling materials (CMC-Na).

The results of the cytotoxicity assays indicate that the administration of CM from SHED on days H1, H3, and H5 can enhance fibroblast and osteoblast viability, particularly with the SCM isolated on day 5 (SCM-H5). This increase in viability is attributed to the presence of various growth factors within the SCM, including TGF-β, VEGF, and HGF, which can induce greater cell proliferation. The results of this study align with the assertion that several biological factors can gradually influence the fluctuations in growth factors within SCM; however, owing to the dynamic nature of SHED cellular behavior, the levels of these secreted growth factors can vary. 40 Owing to the inhibition of interaction when cells are highly confluent, SHED may produce fewer growth factors. The secretion of growth factors may be influenced by nutrient depletion, metabolite accumulation, and waste product in the culture medium. 41 The NESCM-H1, NESCM-H3, and NESCM-H5 groups showed decreased cell viability; however, the percentage of fibroblast and osteoblast viability remained within 60 to 80%. This finding suggests that the NESCM formulation exhibits low toxicity. 42 The increase in the pH level following NESCM formulation preparation may contribute to cell death. The pH level changes in the medium can significantly interfere with cell growth. Cells have optimal pH ranges for their growth and function, and deviations from this range can have various negative effects, including altered enzyme activity, disrupted membrane integrity, impaired nutrient uptake, and increased oxidative stress. 43 The optimal pH range for cell culture varies depending on the cell type. However, most mammalian cells thrive in a slightly alkaline pH range of ∼7.2 to 7.4. In addition, it is essential to choose a gelling agent that remains stable within the specified pH range and exerts little influence on cell proliferation. Some gelling agents are specifically designed to be biocompatible and have minimal side effects on the cells.

The limitation of this study is in the characterization of stem cell and cytotoxicity test. Characterization of stem cells was done using surface marker marking (ICC) and osteogenic differentiation only, without trilineage differentiation. This research made two concentrations of nanoemulgel for the cytotoxicity test: 1.5 and 1.8% NESCM. The 1.8% concentration was not good for attaching cells. The cell floated on the surface of the cell medium. The weight of the gel might have had an effect on the cells when it was added to each well in the NESCM 1.8% group. The suggestion for future research should include more freeze-thaw cycle tests to check for stability, spreadability tests, and viscosity measures.

## Conclusion

To optimize the production and efficacy of SHED-CM, selecting the appropriate primary cell passage is pivotal. SHED-CM at the fourth passage, incubated until day 5 postconfluence, produces growth factors, including TGF-β and VEGF, which are essential for wound healing. A nanoemulgel formulation characterized by both hydrophilic and lipophilic properties can be selected to enhance the penetration and stability of its components, thereby presenting potential for development in biotherapy, especially at NESCM 1.5%.
